# A Toolkit for Orthogonal and *in vivo* Optical Manipulation of Ionotropic Glutamate Receptors

**DOI:** 10.3389/fnmol.2016.00002

**Published:** 2016-02-02

**Authors:** Joshua Levitz, Andrei T. Popescu, Andreas Reiner, Ehud Y. Isacoff

**Affiliations:** ^1^Department of Molecular and Cell Biology, University of California, BerkeleyBerkeley, CA, USA; ^2^Department of Biology and Biotechnology, Ruhr-University BochumBochum, Germany; ^3^Helen Wills Neuroscience Institute, University of California, BerkeleyBerkeley, CA, USA; ^4^Physical Bioscience Division, Lawrence Berkeley National LaboratoryBerkeley, CA, USA

**Keywords:** chemical optogenetics, photo-pharmacology, glutamate receptor, *in vivo*, molecular engineering

## Abstract

The ability to optically manipulate specific neuronal signaling proteins with genetic precision paves the way for the dissection of their roles in brain function, behavior, and disease. Chemical optogenetic control with photoswitchable tethered ligands (PTLs) enables rapid, reversible and reproducible activation or block of specific neurotransmitter-gated receptors and ion channels in specific cells. In this study, we further engineered and characterized the light-activated GluK2 kainate receptor, LiGluR, to develop a toolbox of LiGluR variants. Low-affinity LiGluRs allow for efficient optical control of GluK2 while removing activation by native glutamate, whereas variant RNA edited versions enable the synaptic role of receptors with high and low Ca^2+^ permeability to be assessed and spectral variant photoswitches provide flexibility in illumination. Importantly, we establish that LiGluR works efficiently in the cortex of awake, adult mice using standard optogenetic techniques, thus opening the door to probing the role of specific synaptic receptors and cellular signals in the neural circuit operations of the mammalian brain in normal conditions and in disease. The principals developed in this study are widely relevant to the engineering and *in vivo* use of optically controllable proteins, including other neurotransmitter receptors.

## Introduction

The ability to manipulate neuronal activity using optogenetic tools is a powerful technique for probing synaptic transmission and plasticity, mapping neural circuits, and exploring the cellular basis of behaviors in a variety of organisms (Fenno et al., 2011; Miesenböck, 2011). Most studies rely on expression of non-native light-sensitive proteins, such as opsins, which can either elicit or inhibit action potential firing in genetically defined cells. This method is suitable for probing circuit function and associated behavioral correlates but is limited in its ability to provide insight into the subcellular, molecular events that underlie brain function. Signaling in neurons occurs through numerous mechanisms, which involve a wide variety of proteins including diverse families of ion channels, transporters, G protein-coupled receptors, and enzymes. To probe the molecular basis of the various physiological functions of the nervous system one requires a method to manipulate natively expressed signaling proteins with the spatial, temporal, and genetic precision afforded by optogenetics.

Glutamate serves as the major excitatory neurotransmitter in the central nervous system and exerts its effects primarily via glutamate receptors (GluRs), including both ionotropic (iGluRs) and metabotropic (mGluRs) receptors (Niswender and Conn, 2010; Traynelis et al., 2010). The molecular diversity of GluRs and their overlapping expression patterns make it difficult to fully dissect how individual receptor subtypes contribute to neuronal signaling, behavior, and neurological disorders using classical pharmacological or genetic techniques. For instance, many of the same receptor subtypes are expressed in multiple compartments within the same synapse or multiple cell types within the same brain region. Furthermore, RNA editing, post-translational modifications, accessory subunits and the formation of heteromers further complicate the interpretation of such experiments. Finally, the temporal profile of native GluR activation is difficult to replicate with the coarse control afforded by soluble pharmacological agents or genetic manipulations. For these reasons optochemical approaches, such as caged compounds, have been developed to enhance both the spatial and temporal manipulation of GluRs (Callaway and Yuste, 2002; Ellis-Davies, 2007). While valuable approaches both *in vitro* and *in vivo*, these techniques are limited spatiotemporally by diffusion, limited in pharmacological specificity, and cannot allow one to exert genetic control to target specific cells or to introduce mutations that alter receptor function in a defined way.

We have previously employed a chemical optogenetic approach to optically control individual GluRs based on photoswitchable tethered ligands (PTLs) (Reiner et al., 2015). PTLs are modular photoswitches that site-specifically attach covalently to a protein of interest and reversibly present a functional group via photoisomerization of a light-sensitive moiety, such as azobenzene (Kramer et al., 2013). Even compared to other optical techniques, PTLs allow particularly precise spatiotemporal control since the photoisomerization of azobenzene is a picosecond process and binding is not limited by diffusion. The covalent attachment of the PTL to the target protein provides complete subtype specificity compared to soluble pharmacological agents. Genetic targeting of proteins can allow further precision by pinpointing photocontrol to specific sites within a neural circuit. PTLs of the MAG (Maleimide-Azobenzene-Glutamate) family have been successfully used to engineer light-gated iGluRs, termed LiGluRs (Volgraf et al., 2006; Gorostiza et al., 2007) and mGluRs, termed LimGluRs (Levitz et al., 2013). LiGluR is based on the kainate receptor GluK2 (formerly GluR6), which plays distinct roles at excitatory and inhibitory synapses throughout the brain and has been implicated in a variety of neurological diseases, including mood disorders and epilepsy (Contractor et al., 2011; Lerma and Marques, 2013). This tool has been used to optically manipulate GluK2 activity and, thus, control cellular excitability in cultured neurons and glia (Szobota et al., 2007; Hou et al., 2011; Li et al., 2012), brain slice (Janovjak et al., 2010), and *in vivo* in zebrafish (Szobota et al., 2007; Wyart et al., 2009), fruit fly (Kauwe and Isacoff, 2013), and mouse retina (Caporale et al., 2011; Gaub et al., 2014). PTL-based optical control of GluRs remains to be demonstrated in the mouse brain *in vivo* where it would be a particularly powerful tool for deciphering the roles of GluR signaling in physiology and disease.

The further use of LiGluRs raises a number of challenges related to the engineering and application of such tools. PTL-based approaches provide a high degree of modularity, since receptor and ligand can be changed in a rational and largely independent way that allows one to alter the tool in order to optimize it for the application of interest. By further engineering LiGluRs we can gain more insight into how these tools function pharmacologically and biophysically while gaining a further level of control over receptor function. In addition, a key remaining step for the application of LiGluRs would be the ability to establish optical control *in vivo* in the brain of rodents. This work fully establishes a family of variants of LiGluR with tuneable glutamate affinity, spectral properties, and ion selectivity that functions to control neuronal firing. We also find that LiGluR can be used to manipulate neuronal firing using either dual-color or single-color photoswitches *in vivo* in the cortex of the adult mouse. Ultimately, this work will lead to a finely-tuned molecular optogenetic approach to study the role of specific proteins ranging from the cellular to network to organismal level.

## Materials and methods

### Preparation of photoswitches

Chemical synthesis of L-MAG0, L-MAG1, and L-MAG0_460_ was performed as described previously (Volgraf et al., 2006; Kienzler et al., 2013). Photoswitches were dissolved in dry DMSO (stock concentration ~50 mM) and stored at −20°C.

### Electrophysiological characterization of LiGluRs in HEK cells and cultured hippocampal neurons

LiGluR [rat GluK2(L439C) a isoform] variants were generated by site-directed mutagenesis and used as either untagged versions in a pcDNA3 vector, or tagged with enhanced green fluorescent protein (EGFP) in a pNICE vector. The amino acid numbering includes the wild-type signal peptide. C-terminally EGFP-tagged constructs were used to test for LiGluR localization in cultured neurons and in some current clamp recordings, but did not show any differences in function or expression level compared to untagged constructs.

Whole-cell recordings were performed at room temperature (22–24°C) using an Axopatch 200B headstage/amplifier (Molecular Devices) on an inverted microscope (Olympus IX series). Voltage-clamp recordings were typically performed at −75 mV. Data were analyzed with Clampfit (Molecular Devices) and ProFit (Quantumsoft).

A Xe-lamp light source was used for photoswitching, either a DG4 (Sutter) in combination with excitation filters (379/34 nm and 500/24 nm for regular MAG, 445/20 nm for MAG_460_, “center”/full width >90%) or a Polychrome V monochromator (Till Photonics; 15 nm FWHM bandwidth). A liquid light guide was used for coupling of the light source to the back-port of the inverted microscope and homogeneous epi-illumination was achieved through a 40x LUCPlanFLN NA 0.60 FN 22 objective (Olympus). Neutral density filters (Omegafilters) were used to vary the light intensity, which was measured at the sample stage using a power meter (Thorlabs).

HEK cell recordings were performed as described previously (Reiner and Isacoff, 2014a). Cells were transfected using Lipofectamine 2000 (Invitrogen). A plasmid encoding yellow fluorescent protein (YFP) was co-transfected to yield a fluorescent marker protein. After 24-48 h expression at 37°C, the cells were washed with external solution (see below) and briefly incubated with 0.3 mg/ml concanavalin A (ConA) to suppress ligand-induced desensitization. Labeling was performed for 40 min at room temperature using ~50 μM MAG in external solution and the cells were thoroughly washed with external solution to remove any unreacted MAG. The extracellular solution contained (in mM): 138 NaCl, 1.5 KCl, 1.2 MgCl_2_, 2.5 CaCl_2,_ 10 glucose, 10 HEPES, pH 7.3. Patch pipettes were pulled from borosilicate glass to give 3-7 MΩ resistance, when filled with internal solution containing (in mM): 135 K-gluconate, 10 NaCl, 10 HEPES, 2 MgCl_2_, 2 MgATP, 1 EGTA, pH 7.4.

The apparent glutamate affinity (EC_50_) of different GluK2 variants (**Figure 3A**) was determined by obtaining dose response-curves in whole-cell recordings from HEK cells in the presence of ConA. A Hill-type equation:

(1) $$I=I_{\max}*\left(\frac{{\left[\text{Glu}\right]}^{n}}{{\text{EC}}_{50}^{n}+{\left[\text{Glu}\right]}^{n}}\right)$$

was fitted to data from individual cells and subsequently used to calculate the relative current amplitudes. The average of this data is reported in **Figure 3A**, along with a fit of Equation (1) to this average. For GluK2 (E738D), which was not fully saturated with 30 mM glutamate, we slightly modified this procedure. Here we first averaged the titration data obtained from four independent cells by normalizing the currents relative to the current obtained with 10 mM glutamate. This average is reported in **Figure 3A** and subsequent fitting of Equation (1) yielded the corresponding parameters. Normalizing the data to 3 or 30 mM glutamate yielded similar results.

Dissociated hippocampal neuron cultures were prepared from postnatal P0 or P1 mice on 12 mm glass coverslips as previously described (Levitz et al., 2013). Neurons were transfected with GluK2 (L439C) or GluK2 (L439C, K487A) (1.5 μg/well) and tdTomato (0.25 μg/well as a transfection marker) using the calcium phosphate method at DIV9. Whole cell patch clamp experiments were performed 3–6 days after transfection (DIV 12-15). Labeling was performed for 40 min at room temperature using ~50 μM MAG in labeling solution containing (in mM): 150 NMDG, 2.5 KCl, 0.5 CaCl_2_, 5 MgCl_2_, 5 glucose, 10 HEPES, pH 7.5. Recordings were performed in extracellular solution containing (in mM): 138 NaCl, 1.5 KCl, 1.2 MgCl_2_, 2.5 CaCl_2_, 10 glucose, 5 HEPES, pH 7.4. Glass pipettes of resistance 5–10 MΩ were filled with an intracellular solution containing (in mM): 140 K-gluconate, 10 NaCl, 5 EGTA, 2 MgCl_2_, 1 CaCl_2_, 10 HEPES, 2 MgATP, and 0.3 Na_2_GTP, pH 7.2. Current was injected to compare the optical depolarization from a standard potential of −60 mV (**Figure 3F**). Confocal imaging of GFP-tagged LiGluR constructs was performed on a Zeiss LSM780 AxioExaminer.

### Virus production and expression

AAV production was carried out using standard methods (Grieger et al., 2006). Either AAV2 or AAV7M8 (Dalkara et al., 2013) capsids were used to package LiGluR and GFP viruses. Mice were sacrificed ≤3 weeks after injection to assess expression. For immunohistochemistry mice were perfused with 4% paraformaldehyde and 100 μM slices were prepared and stained with a monoclonal anti-GluK2/GluK3 antibody (Millipore) and imaged with a Zeiss LSM780 AxioExaminer confocal microscope. Due to the poor labeling of native receptors with the anti-GluK2/GluK3 antibody (not shown), we were only able to observe heterologously over-expressed LiGluR above background levels.

### Surgical procedures

For all surgical procedures, mice were anesthetized and maintained with a 1.5% isoflurane and oxygen mixture. A craniotomy was made above the target region (V1) using stereotaxic coordinates relative to lambda (0.8 mm anterior, 2.2 mm lateral), and 0.5–1 μl of viral particles (~10^13^–10^14^ viral genomes/ml) were deposited at a depth of 0.8 mm below the surface of the brain using a Nanoject system (Drummond). Additionally, some mice were fitted with small screws attached to the skull with dental acrylic (Teets, AM Systems), for subsequent head-fixing in a custom made setup. The area was then covered with a silicon sealant (Kwik-Cast, WPI).

For electrophysiological recordings mice underwent a second surgical procedure (3–6 weeks later) during which 1 μl of 100 μM L-MAG0 or L-MAG0_460_ was injected at the same V1 location. A custom made movable optrode (similar to Anikeeva et al., 2012) was positioned at the brain surface, and fixed with dental acrylic. Mice were allowed to recover for at least 3 h prior to the recording session.

### *In vivo* optical manipulation and electrical recording

The mouse was head-fixed with a custom made metal piece (eMachineShop, NJ) able to accommodate the screws implanted during surgery, and connected to a breadboard through post holders (Thorlabs). The body of the mouse was placed in an acrylic tube (McMaster-Carr), and the entire setup was housed in a sound-attenuated box.

Optical stimulation was achieved with a custom dual laser system consisting of a 375 nm diode laser (16 mW, Coherent) and a 532 nm DPSS laser (50 mW, Laserglow Technologies). The free laser beams were combined using a 405 nm dichroic mirror (Semrock) and focused into a single optical fiber using a 10x objective (NA 0.25, *f* = 16.5 mm, Newport) mounted on a fiber coupler (F91-C1-T, Newport). Single wavelength stimulation was performed with a fiber-coupled 472 nm laser (Shanghai Laser & Optics Century Co.). In all cases, light was delivered through a 200 μm multimode fiber (NA 0.39, Thorlabs) coupled to ceramic ferrules (Precision Fiber Products Inc.). The intensity of the laser was adjusted based on neuronal responses, and typically resulted in a total power at the end of the fiber of 1–4 mW. Laser illumination was controlled by a computer with a PCI-DAQ board (National Instruments) and custom software developed in Matlab (Mathworks, software freely available at https://github.com/andpopes/LaserStim.git). Neuronal activity was recorded with a movable optrode, consisting of a 200 μm optic fiber surrounded by up to 16 single wire electrodes (California Fine Wire). The construct was similar to Anikeeva et al. (2012), and allowed precise sampling of electrical activity and optical stimulation across various depths. Signals were amplified with a 32-channel TDT RZ5 unit (Tucker-Davis Technologies), digitized at 25 kHz and stored on a computer.

Prior to recordings, each mouse was gradually habituated to head-fixing (5–45 min) over a period of 5 days. On the experimental day mice underwent a second surgical procedure for L-MAG injection (see above), at the end of which the optrode construct was positioned just below the cortical surface. The neuronal responses to various laser stimulation patterns were sampled at depths spanning the entire cortical layer (~1 mm), in 100 μm steps over a period of ~1 h. Only one recording session was performed for each mouse. In some cases, optrodes were advanced only halfway, and the procedure resumed 24 h later.

Single units were identified off-line using principal component analysis with custom software written in Matlab. To identify units significantly modulated by the activation of LiGluRs we measured the firing rates during the first second of laser stimulation, and calculated the fold-change from baseline (1 s prior to laser):

$$\begin{array}{l} \text{Fold change}=(\text{FR during UV}-\text{FR during baseline})/\\ \text{ FR during baseline}, \end{array}$$

where FR is the firing rate. A unit was considered responsive if the fold change was consistently higher (or lower) than 99.9% of values similarly computed with shuffled spikes (10,000 repeats).

### Animal research

All mouse experiments were performed with approval of the University of California Animal Care and Use Committee. The wt mice (C57BL/6J) were purchased from the Jackson Laboratory and the age of the mice ranged from p50–p80 for rAAV injections and from p80–p120 for *in vivo* experiments.

## Results

### Tuning the LiGluR response with MAG photoswitch variants

**Figure 1:**
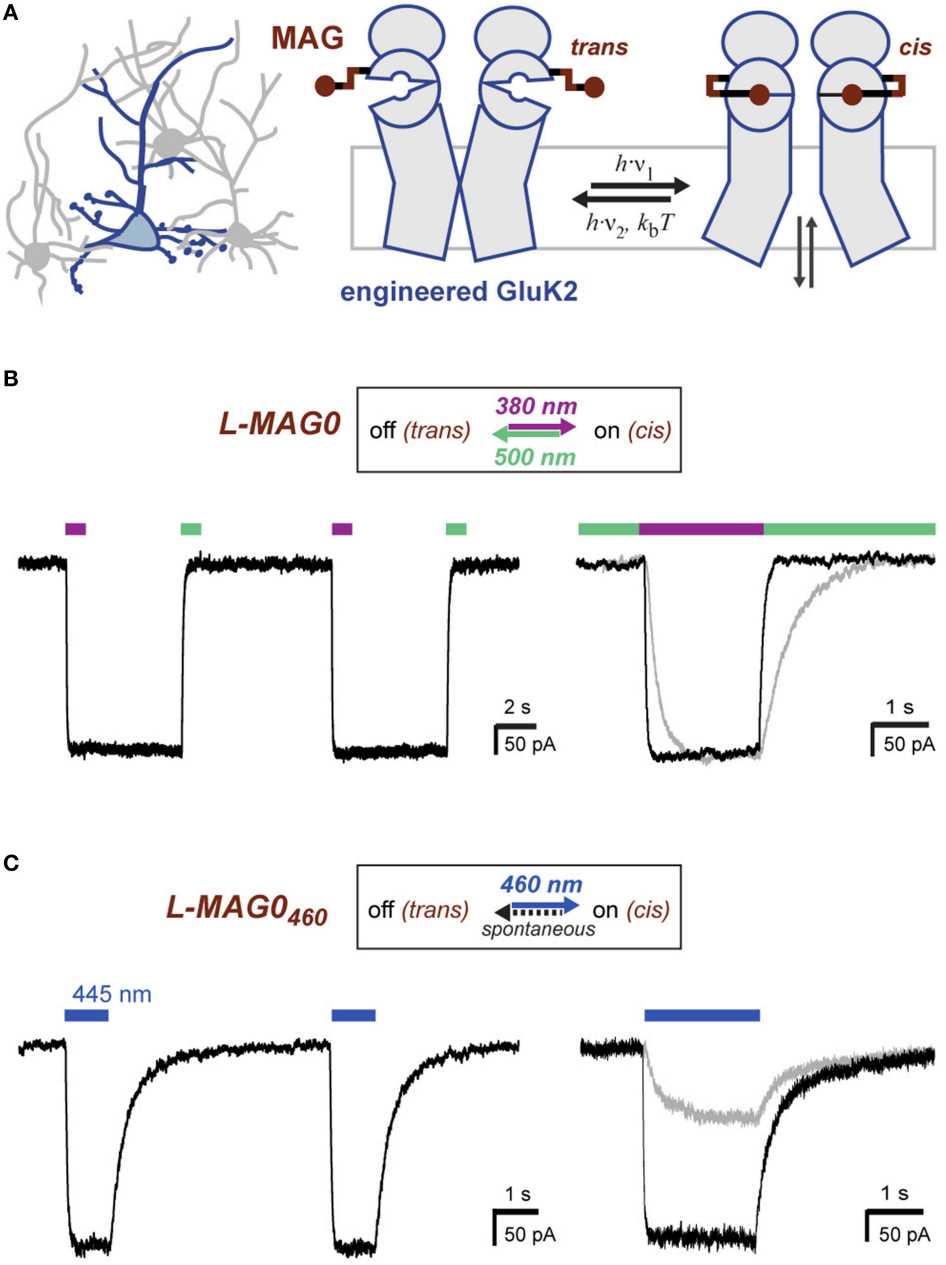
**Operating principles of light-gated glutamate receptors (LiGluRs). (A)** LiGluRs are optogenetic tools that can be expressed in genetically targeted cells (blue). They are based on an engineered iGluR subunit, which is expressed in the desired cell type and conjugated with a synthetic photoswitchable ligand, called MAG. Photoisomerization of the covalently bound MAG ligand from *trans* to *cis* presents the glutamate moiety to the glutamate binding site, which leads to ligand binding and ion channel opening. **(B)** Photoswitching of LiGluR labeled with a regular, bistable MAG ligand (Volgraf et al., 2006; Gorostiza et al., 2007). Illumination with 380 nm light (violet bar) leads to an inward current as shown in a voltage-clamp recording of a LiGluR-expressing HEK cell labeled with L-MAG0. The receptor activation is sustained in the dark and turned off by illumination with 500 nm light (green bar). (Left) Switching is fully reversible as demonstrated with two consecutive switching cycles. (Right) Lowering the light intensity leads to slower photo-activation and deactivation kinetics, but the same current amplitude (black trace ~7-8 mW/mm^2^; gray trace ~0.7-0.8 mW/mm^2^; see also Figure S2). **(C)** Photoswitching of LiGluR labeled with L-MAG0_460,_a blue light activated photoswitch with a fast spontaneous *cis*-to-*trans* relaxation (Kienzler et al., 2013). (Left) HEK cell voltage-clamp recording showing two switching cycles with 445 nm light (blue bar). Once the blue light is turned off, LiGluR turns off spontaneously. (Right) Lowering the light intensity results in slower activation kinetics and a decreased response (black trace ~1.5 mW/mm^2^; gray trace ~0.1 mW/mm^2^). HEK cell recordings were performed in the presence of ConA.

Direct optical control of glutamate receptors is achieved by covalently attaching a PTL to the receptor of interest. LiGluR is a light-gated ionotropic glutamate receptor that is based on the kainate receptor GluK2, which is expressed with a cysteine substitution (L439C) close to the ligand binding site and conjugated with a PTL of the L-MAG family (Figure 1A; Figure S1) (Volgraf et al., 2006; Gorostiza et al., 2007). Once the MAG ligands are attached to the receptor *via* a maleimide-cysteine linkage, they can activate GluK2 in response to light, which isomerizes the MAG azobenzene moiety from *trans* to *cis*, reversibly presenting the glutamate headgroup to the ligand binding domain (Figure 1A). Deactivation is achieved by isomerizing the MAG ligand back to its *trans* configuration, retracting the ligand from its binding site. The glutamate headgroup of L-MAGs resembles (2*S*,4*R*)-4-methylglutamate (SYM 2081), a high-efficacy agonist with some selectivity for kainate receptors (Zhou et al., 1997; Traynelis et al., 2010). The relative efficacy of the ligand in the two photoswitch conformations can be modulated by varying the MAG linker length (L-MAG0, 1 and 2; Figure S1; Numano et al., 2009).

The response of LiGluR to light is largely determined by the photophysical properties of the MAG photoswitch. Chemical substitutions to the azobenzene core allow tuning of the spectral sensitivity and of the *on*/*off* characteristics of LiGluR in a rational manner (Gorostiza et al., 2007; Kienzler et al., 2013; Izquierdo-Serra et al., 2014; Rullo et al., 2014) (Figure 1). For *in vivo* applications, two aspects are particularly important: First, regular MAGs offer bistability: light of 370–405 nm leads to receptor activation with sustained activation in the dark, but a second wavelength of light, >480 nm, is needed to turn LiGluR off (Figure 1B), since the spontaneous *cis*-to-*trans* relaxation of regular MAGs is slow (tens of minutes; Gorostiza et al., 2007). Because of this, we predict a second property, which is useful for *in vivo* applications and which we demonstrate here. We had previously shown that high light intensities can be used to control LiGluR with submillisecond time resolution to mimic the relevant synaptic timescales and produces the fast and pronounced desensitization typical for kainate receptors (Reiner and Isacoff, 2014b). We now show that maximal photo-activation is independent of light intensity (Figure 1B, gray line). Lowering the light intensity slowed activation and deactivation, but resulted in the same current amplitude, as expected for a system with a very slow spontaneous back-rate (Figure 1B, gray line).

In contrast, LiGluR-MAG_460_ (GluK2 (L439C) + L-MAG0_460_) is activated with visible light (maximal activation with blue, 460 nm light) and spontaneously relaxes back to the *trans* configuration within less than a second in the dark (Kienzler et al., 2013) (Figure 1C). We find lower light intensities to both slow activation and result in a smaller amplitude photo-current (Figure 1C, gray line), as predicted for a system with a substantial spontaneous back-rate (Gaub et al., 2014). Thus, intensity can be used to tune the amplitude of LiGluR-MAG0_460_ photo-current.

**Figure 2:**
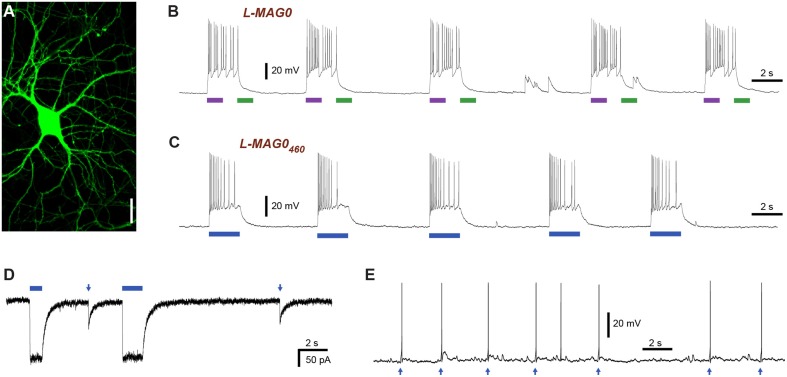
**Light-induced depolarization and optical control of neuronal firing with LiGluR. (A)** LiGluR-GFP expression in a cultured hippocampal neuron visualized with confocal imaging. Scale bar = 20 μm. **(B)** LiGluR labeling with conventional L-MAG0 allows for optical depolarization in a bistable fashion: 375 nm light (violet bar) depolarizes the cells, which results in an increase in action potential firing, illumination with 480 nm light (green bar) readily reverses the effect (current-clamp recording at V_rest_ = −60 mV). **(C–E)** LiGluR labeled with L-MAG0_460_ allows optical control of neuronal firing with light of a single, blue wavelength. **(C)** The effect of 445 nm illumination (blue bar) reverses spontaneously in the dark (current-clamp recording at V_rest_ = −57 mV). **(D)** Voltage-clamp recording at −60 mV showing inward currents resulting from long (seconds) and short (5 ms) 445 nm light pulses. **(E)** Short 445 nm light pulses (arrows; 3 ms) are sufficient to elicit single action potentials (current-clamp recording at U_base_ = −47 mV).

LiGluR expresses well in cultured neurons where it traffics throughout fine processes (Figure 2A). Since GluK2 is a nonspecific cation channel that natively contributes to the generation of excitatory postsynaptic currents (Lerma, 2003) LiGluR depolarizes neurons in a light-dependent manner (Szobota et al., 2007). Current-clamp recordings of cultured hippocampal neurons expressing LiGluR revealed robust depolarization in a bistable and fully reversible fashion, which is sufficient to induce reproducible bouts of action potential firing (Figure 2B). Importantly, we found that LiGluR-MAG0_460_ induces comparable depolarizations at blue light intensities typical for optogenetic experiments (~1 mW/mm^2^). As expected for LiGluR-MAG0_460_, turning off the light leads to spontaneous deactivation and a return to the baseline potential (Figure 2C). Pulses of 445 nm light (~1 mW/mm^2^) were sufficient to induce large inward currents in voltage-clamp mode (Figure 2D) and brief (3 ms) pulses of light were found to be sufficient to elicit single action potentials (Figure 2E). This demonstrates that L-MAG0_460_ provides a powerful addition to optical control for kainate receptors *in vivo*, which provides the advantage of requiring only light of a single, blue wavelength, thereby leaving the rest of the visible spectrum for other optical manipulation or detection tools.

### Tuning the physiological function of LiGluR by receptor engineering

Apart from its optical controllability, LiGluR resembles native GluK2 receptors in all key aspects, displaying normal glutamate affinity (Gorostiza et al., 2007) as well as normal desensitization and recovery properties (Reiner and Isacoff, 2014b). Moreover, pharmacological antagonists, such as DNQX, are still able to block the response to glutamate or MAG photoswitching (Gorostiza et al., 2007). It should also be noted that photoactivation can be performed on the synaptic (i.e., submillisecond) timescale and that photo-activation results in the fast and pronounced desensitization typical for kainate receptors (Reiner and Isacoff, 2014b). The conservation of native GluK2 properties provides an opportunity to further engineer LiGluR based on known principles of receptor function in order to make designer variants for specific applications.

When LiGluR is introduced in neurons it will, presumably, contribute to neuronal signal transmission like any other GluK2 subunit and will respond to endogenous glutamate released from presynaptic neurons. This is favorable in some scenarios, particularly after a knock-in of the L439C mutation into the native receptor gene, but may be less desirable in other cases, in which LiGluR is expressed ectopically. A fully orthogonal LiGluR, meaning a receptor that is only activated by light but not by glutamate, would also be useful to probe the role of this receptor against the background of normal glutamatergic transmission. We therefore attempted to place additional mutations in LiGluR to lower its affinity for glutamate, while maintaining robust expression and high enough affinity to respond to MAG. We hypothesized that this is plausible since MAG, as a covalently attached ligand, is present at a high effective concentrations (>10 mM) when isomerized to the *cis* configuration (Gorostiza et al., 2007).

**Figure 3:**
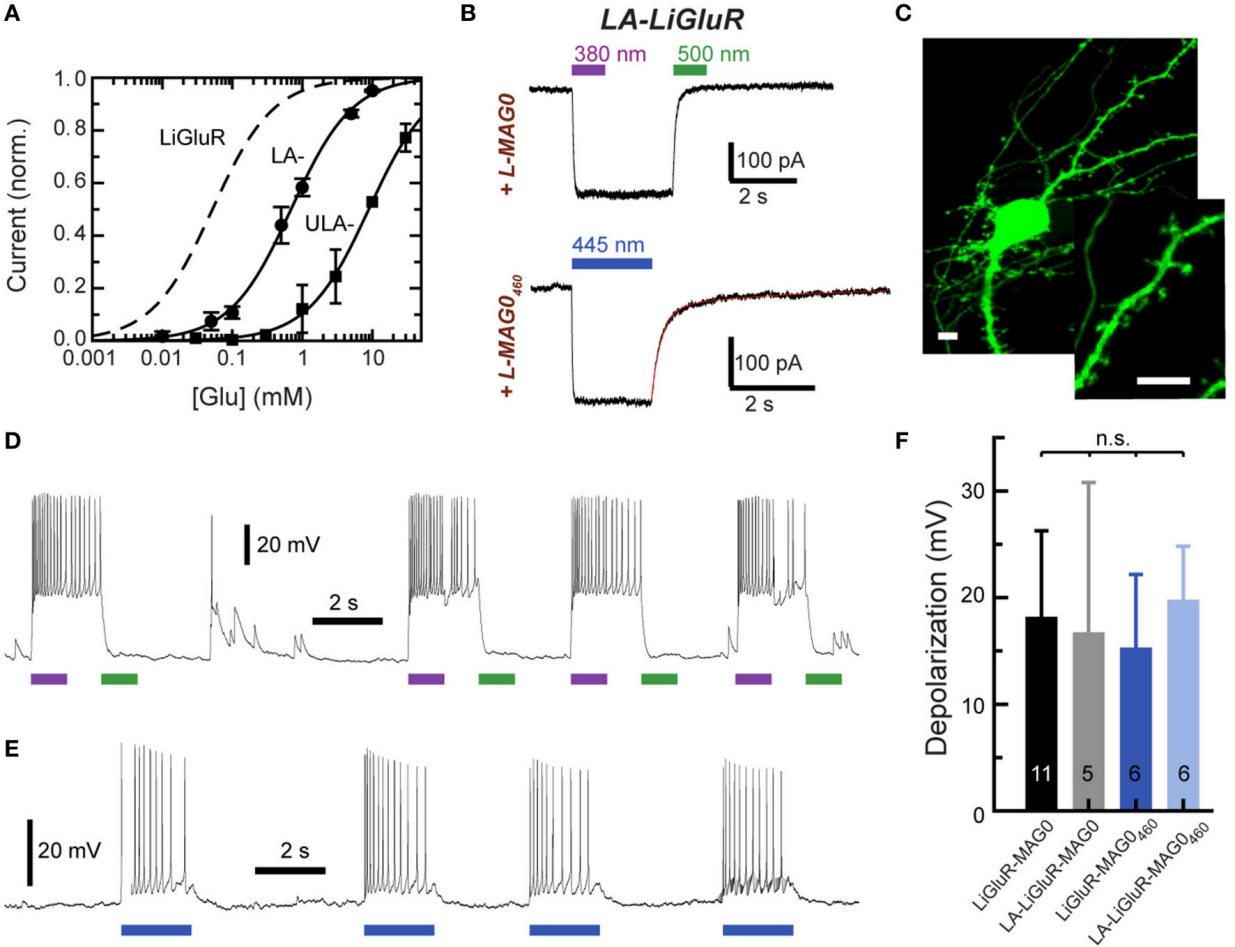
**Engineering and characterization of LiGluR variants with low glutamate affinity. (A)** Glutamate dose-response properties of LiGluR, LA-LiGluR [LiGluR(K487A)] and ULA-LiGluR [LiGluR(E738D)] obtained from HEK cell voltage-clamp recordings in the presence of Con A. Apparent affinities were determined by fitting with a Hill-type equation, which yielded EC_50_ = (0.70 ± 0.16) mM (Θ = 1.00± 0.07, *n* = 3 cells) for LA-LiGluR, and EC_50_ = (10.0 ± 2.6) mM (Θ = 0.95 ± 0.10, *n* = 4 cells) for ULA-LiGluR. Data points report the mean ± s.d. LiGluR was reported to have a glutamate EC_50_ = (52 ± 1) μM (from Gorostiza et al., 2007). **(B)** Photoactivation of LA-LiGluR after labeling with regular L-MAG0 (top) and L-MAG0_460_ (bottom). Voltage-clamp recordings in the presence of ConA with DG4 illumination (1–2 mW/mm^2^), **(C)** Confocal image of hippocampal neurons expressing LA-LiGluR-EGFP. Scale bars = 10 μm. **(D)** Optical control of hippocampal neurons using LA-LiGluR-EGFP labeled with regular L-MAG0 (current-clamp recording at V_rest_ = −76 mV), or, **(E)** labeled with L-MAG0_460_ (current-clamp recording at V_base_ = −40 mV). **(F)** Summary of light-induced depolarization achieved with different combinations of LiGluR and MAG from a common potential of −60 mV (mean ± s.d. with number of cells). An ANOVA test did not detect significant differences between the groups (n.s.).

Two mutations turned out to be particularly useful for lowering the glutamate affinity of LiGluR. The first of these was at K487, an amino acid that is located on the tip of the ligand binding cleft, which stabilizes the closed conformation of the ligand binding domain that is thought to cause receptor activation. As previously reported (Weston et al., 2006), the mutation of this residue to alanine (K487A) reduced the sensitivity of LiGluR to glutamate ~20-fold, resulting in an apparent EC_50_ of (0.70 ± 0.16) mM in the presence of concanavalin A (ConA) (Figure 3A). LiGluR (K487A), which we named “low-affinity LiGluR” (LA-LiGluR), could be labeled and optically controlled with L-MAG0, yielding robust photocurrents (Figure 3B, top). LA-LiGluR could also be photo-controlled with L-MAG0_460_ (Figure 3B, bottom). This suggests that despite subtle differences in chemical structure (Figure S1), this MAG variant is also able to present the glutamate headgroup at a high effective concentration in the *cis* state. The spontaneous relaxation properties of L-MAG0_460_ were not affected by the reduced affinity of LA-LiGluR (Figure S3), suggesting that deactivation is rate-limited by thermal relaxation in the dark of L-MAG0_460_ back to the more stable *trans* state, rather than by the binding energy of the glutamate in the binding pocket.

We found that LA-LiGluR expresses well in neurons, traffics into distant dendritic branches as well as spines, and that it can be used to efficiently control neuronal firing (Figures 3C–F). A direct comparison of LiGluR and LA-LiGluR combined with either L-MAG0 or L-MAG0_460_ revealed no significant differences in the capacity to depolarize hippocampal neurons (Figure 3F). Due to its low affinity, LA-LiGluR should not respond to endogenous baseline levels of glutamate, although it may respond weakly to synaptic glutamate. We therefore next sought to decrease the glutamate affinity of LiGluR even further to make a fully orthogonal receptor that is controlled by light only and no longer responds to physiological levels of glutamate.

To further lower the affinity of LiGluR we turned to the glutamate to aspartate mutation at residue 738 (E738D), which has been shown to result in an extremely low glutamate affinity (Mah et al., 2005). This amino acid is directly positioned in the glutamate binding pocket. The apparent EC_50_ of LiGluR (E738D) was estimated to be 10.0 ± 2.6 mM in the presence of ConA, that is ~200-fold lower than wild type, leading us to name LiGluR (E738D) ultra-low affinity LiGluR (“ULA-LiGluR”) (Figure 3A). This affinity is so low that ULA-LiGluR is not expected to respond even during peak glutamate in the synaptic cleft following transmitter release. Despite its ultra-low affinity, ULA-LiGluR expressed well and could be photo-controlled with L-MAG1 (Figure S4A). Interestingly, the slightly shorter L-MAG0 was unable to optically manipulate this variant (data not shown). Together, these experiments show that the local concentration of MAG is high enough to overcome decreases in apparent glutamate affinity of up to 200-fold, while maintaining fast photo-activation and deactivation.

An important and distinguishing characteristic of iGluRs is their variable selectivity for monovalent vs. divalent cations, which results in different Ca^2+^ permeabilities. GluK2 can undergo RNA editing at a key pore-lining residue that controls its permeability to Ca^2+^ (Egebjerg and Heinemann, 1993; Dingledine et al., 1999). LiGluR is based on the unedited GluK2(Q) form (Q621), which shows substantial Ca^2+^ conductance (Burnashev et al., 1995). LiGluR photo-activation hence leads to a significant influx of Ca^2+^ (Volgraf et al., 2006; Izquierdo-Serra et al., 2013; Carroll et al., 2015), which acts as a 2nd messenger that can trigger intracellular signaling events and changes in synaptic plasticity. We sought to make a version of LiGluR [“LiGluR(R)”] based on the edited form (Q621R) to reduce Ca^2+^ influx (Burnashev et al., 1995). We found that LiGluR(R) maintains robust L-MAG0 photoswitching (Figure S4B). This now provides us with the ability to optically control both the high and low Ca^2+^ permeability versions of GluK2 receptors for the analysis of their roles at synapses, as well as to produce membrane depolarization with high and low Ca^2+^ influx. Ultimately, comparison of both the edited and un-edited versions of LiGluR can allow one to probe the role of Ca^2+^ vs. monovalent ion influx in physiological functions.

In summary, these results highlight the modularity of LiGluR, where changes can be made to the photoswitch to tune the photo-response and to the receptor to change its physiological action.

### Optical control of LiGluR in mouse cortex *in vivo*

Having established a family of LiGluR variants with a range of properties, we next turned to the intact mammalian brain. While fundamental work on glutamate receptor neurobiology may be done in simplified *in vitro* or *ex vivo* systems, as well as in model organisms, rodents offer the ability to control specific proteins within the intact circuits of a mammalian brain. Controlling specific GluRs *in vivo* in mice using PTLs opens the door to probing specific GluR function in physiological, behavioral and pathological contexts with previously unattainable precision.

**Figure 4:**
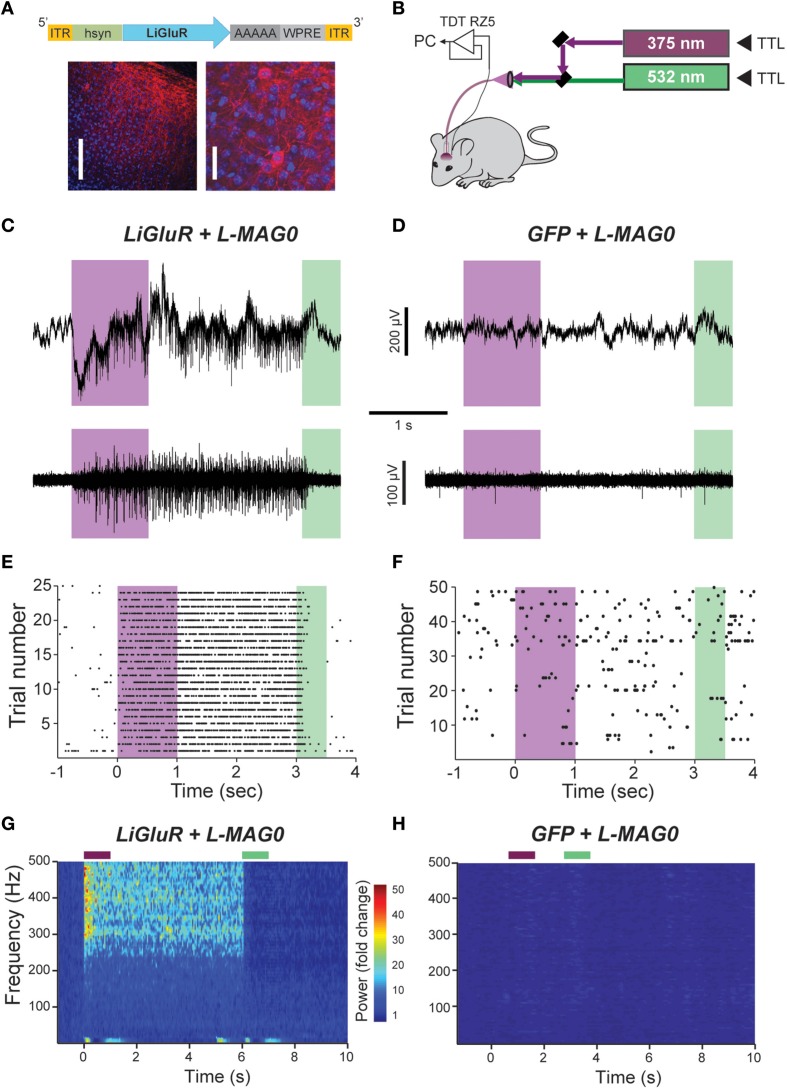
*****In vivo*** optical control of LiGluR in the visual cortex of awake mice. (A)** Top, map for AAV vector used to produce AAV-hsyn-LiGluR. Bottom, expression of GluK2/LiGluR in V1 neurons as visualized by immunohistochemistry. Scale bars = 200 μm, left and 10 μm, right. **(B)** Schematic showing experimental setup of *in vivo* electrophysiology experiments. A TTL-controlled dual laser system was connected to an optrode which was implanted into V1 of an awake, head-fixed mouse and was attached to a TDT-Rz5 amplifier. **(C,D)** Representative recordings from either a LiGluR **(C)** or GFP **(D)** injected mouse following L-MAG0 injection. Top, unfiltered recordings of electrical activity in response to 375 nm (violet) or 532 nm (green) illumination. Bottom, high-pass filtered electrical recordings. **(E,F)**, Representative raster plots for individual units showing repeatability of LiGluR photoactivation **(E)** and no response in neurons not infected with LiGluR **(F)**. **(G)** Local field potential (LFP) responses to LiGluR activation were observed for L-MAG0. Data is presented as normalized power (z-axis, color coded) as a function of time (x-axis) and frequency (y-axis). Power was normalized to the 1 s period prior to laser stimulation for each frequency. **(H)** No LFP response was observed in GFP-injected mice.

**Figure 5:**
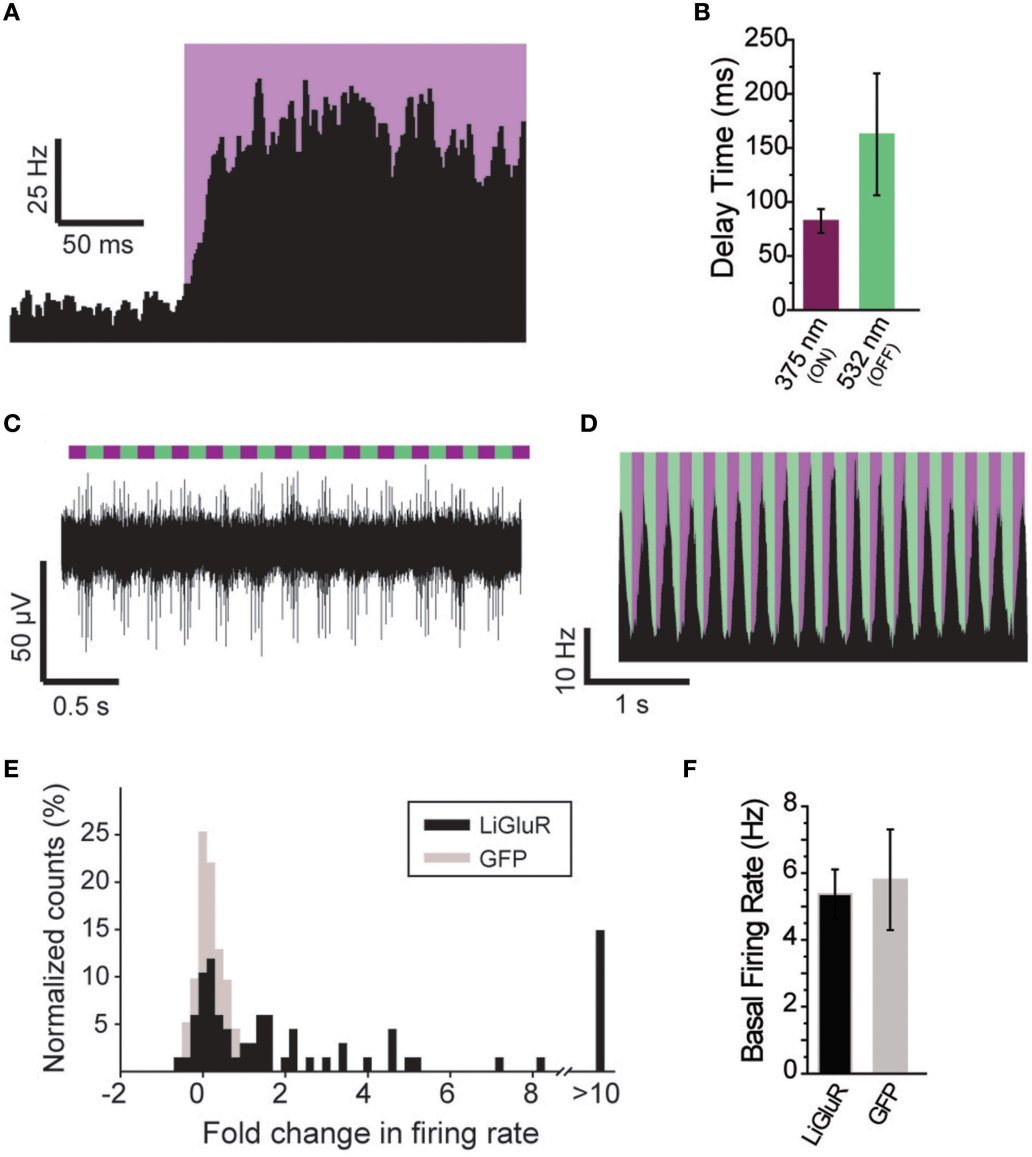
**Further characterization of ***in vivo*** optical control of LiGluR. (A)** Summary of light response from a representative cell showing the speed of response. **(B)** Kinetics for the activation (purple) and inactivation (green) of LiGluR with L-MAG0. Values represent the delay between the onset of laser stimulation and the earliest change in firing rate, averaged across all single units recorded. Data is presented as mean ± s.e.m. **(C**) Representative filtered recording from V1 of a LiGluR-expressing and L-MAG0-labeled mouse showing high frequency light responses. **(D)** Summary of firing rate changes in a representative cell in response to high frequency photoswitching. **(E)** Summary histogram for all units recorded from either LiGluR (black) or GFP (gray) -infected mice. **(F)** Baseline firing rates (y-axis, mean ± s.e.m.) for periods between laser stimulations, in mice expressing LiGluR or GFP. No difference was observed between the two groups.

The ability to control neuronal activity with LiGluR *in vivo* was tested with common techniques, by combining viral delivery of LiGluR with local fiber-mediated optical stimulation and extracellular recordings (Anikeeva et al., 2012). We expressed LiGluR in the V1 area of the visual cortex of adult mice (8–10 weeks old) using an adeno-associated virus (AAV) under the control of the hsyn promoter to broadly target neurons (Figure 4A, top). GluK2 is natively expressed throughout the neocortex where it modulates cell and network excitability through a variety of mechanisms (Petralia et al., 1994; Contractor et al., 2011; Lerma and Marques, 2013). Three to 6 weeks following AAV injection provided sufficient time for detection of added GluK2 expression around the injection site (Figure 4A). Following this period, L-MAG0 was injected and an optrode was implanted at the same injection site. After >3 h of post-surgical recovery, mice were connected through an optical fiber to a dual laser illumination system (Figure 4B), and neuronal activity was monitored. As expected for optical activation of GluK2 in neocortex, brief illumination with 375 nm light produced a rapid increase in neuronal firing that was stable in the dark, and reversed by 532 nm illumination (Figure 4C). Repeated photoactivation and deactivation cycles produced a highly repeatable modulation of firing in individual cells (Figure 4E). In contrast, control mice injected with a GFP virus, followed by the same delayed L-MAG0 injection and optrode placement did not show any light response (Figures 4D,F). Measurement of local field potential (LFP) confirmed the increase in neuronal activity induced by LiGluR activation (Figure 4G), but not in MAG-injected, GFP-infected mice (Figure 4H). LiGluR-induced optical manipulation of firing rates occurred with post-illumination delays as short as ~30 ms with averages in the 70–150 ms range as expected for the light intensity used in this experiment (Figures 5A,B). The repeatability and rapid kinetics of LiGluR photoactivation allowed entrainment of neuronal firing at frequencies up to 5 Hz (Figures 5C,D). Figure 5E shows a summary of all recorded cells in both LiGluR and GFP mice. In LiGluR injected mice (*n* = 4), activation with 375 nm light induced a significant increase in the firing rate of 60% of recorded neurons (with 7% showing a >10-fold increase), and a significant decrease in 4% of neurons (see methods). In contrast, similar light stimuli did not elicit this effect in GFP-injected mice (*n* = 3; 0% of neurons showed an increase, 2% showed a decrease in firing rates). Chi-squared analysis revealed a significant difference between the distribution of non-responsive, up and down- regulated firing rates for the two groups (Chi^2^ = 11.53, *df* = 2, *p* = 0.003). Baseline firing rates of recorded neurons varied greatly in both groups (values between 0.7 and 23 Hz), consistent with a pan-neuronal viral transfection. Furthermore, we did not observe any significant difference between baseline firing rates in the experimental vs. control groups indicating that MAG labeling and LiGluR expression did not alter the basal firing rates of the region (Figure 5F).

**Figure 6:**
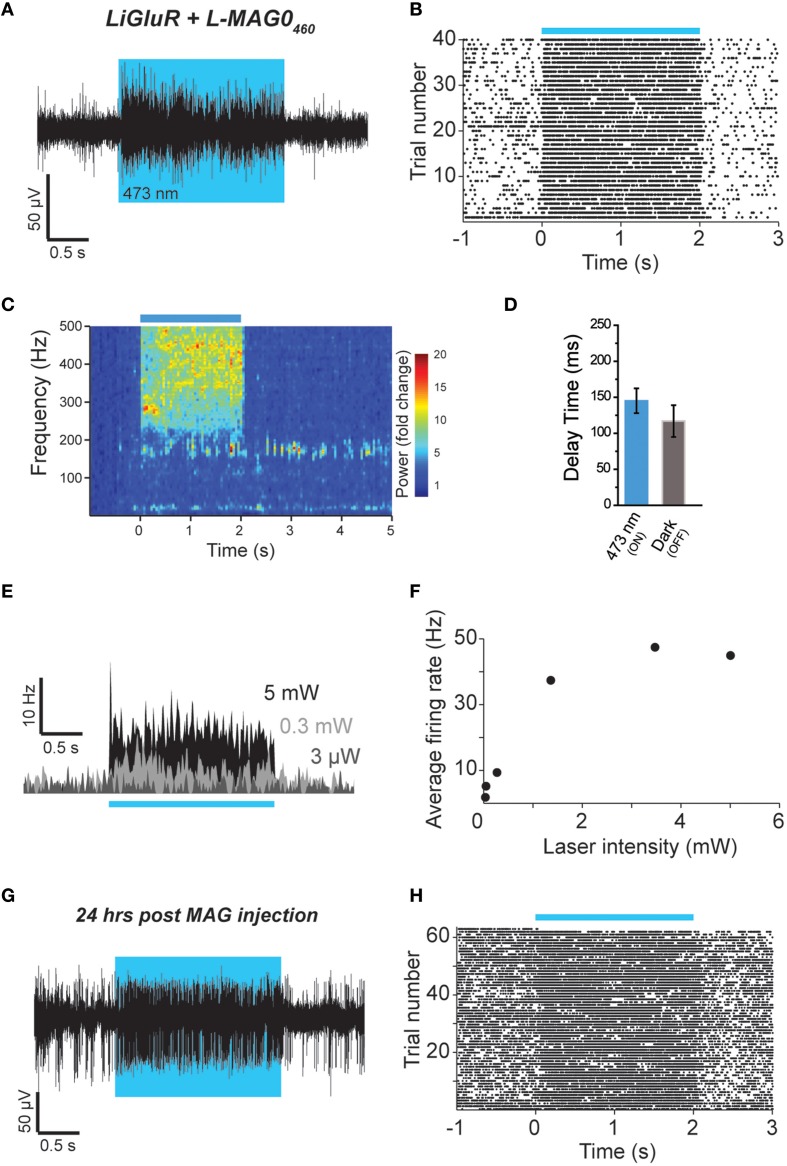
**Single wavelength optical control of LiGluR ***in vivo*** with L-MAG0_460_. (A,B)** LiGluR activation with L-MAG0_460_ results in increased firing in response to 473 nm light that is rapid, repeatable and spontaneously-reversed in the dark. **(C)** LFP changes are observed in response to LiGluR activation with L-MAG0_460_. **(D)** Kinetics for the activation (blue) and inactivation (gray) of LiGluR using L-MAG0_460_. **(E)** Representative recording showing power-dependence of LiGluR photoactivation with L-MAG0_460_. **(F)** Summary of power-dependence of photoactivation for a representative cell. **(G,H)** 24 h post MAG injection light responses to 473 nm light were maintained.

We next tested L-MAG0_460_ *in vivo* to establish single wavelength optical control of LiGluR in mouse cortex. Following infection with AAV-LiGluR and injection of L-MAG0_460_ in V1 (*n* = 2), 473 nm illumination produced a rapid and repeatable increase in neuronal firing that was reversible in the dark (Figures 6A,B) on a similar time scale to cultured cell experiments (Figure 1C). Similar to L-MAG0, L-MAG0_460_ activation led to a strong LFP response in V1 (Figure 6C). L-MAG0_460_-mediated photoactivation of LiGluR resulted in manipulation of firing rates on similar time scales to L-MAG0 (Figure 6D). Consistent with the pan-neuronal expression of the hsyn promoter, we observed occasional inhibitory light responses, presumably from feed-forward inhibition (Figure S5). Unlike L-MAG0, which populates a wavelength-dependent photostationary state that is light intensity-independent, titration of laser intensity led to a tuneable response in V1 neurons labeled with L-MAG0_460_ (Figures 6E,F). Finally, we wondered if a single injection of MAG molecules could allow for optical control of LiGluR over a longer time period than hours following injection. 24 h after injection of L-MAG0_460_ in V1 we still observed robust photoactivation of LiGluR-expressing neurons (Figures 6G,H), indicating that MAG labeling and LiGluR surface expression is stable for at least a day following injection. This is consistent with recent work in the retina showing LiGluR-mediated light responses for up to 2 weeks following injection (Gaub et al., 2014) and indicates that LiGluR may be used *in vivo* for behavioral assays that last for days rather than hours.

## Discussion

In this study, we establish for the first time that LiGluR may be used *in vivo* in the neocortex of adult mice in conjunction with fiber-based optogenetic technologies to allow for rapid optical control of GluK2 activity. AAV-mediated expression of LiGluR in visual cortex was found to produce sufficient levels of surface GluK2 in cortical neurons after ~3 weeks. MAG labeling occurs rapidly following injection, shows no gross adverse effects on animal or tissue health, permits selective photoactivation of engineered receptors within a pool of native receptors, and remains bound to LiGluR for at least 24 h post-labeling. This tremendously expands the applicability of MAG photoswitches to control LiGluRs (and likely LimGluRs), which have already found successful application in brain slices, flies and zebrafsh larvae, as well as the retina of mice and dogs (Reiner et al., 2015). Our work complements previous studies in cell culture that have shown that LiGluR expression and labeling does not alter cell health (Szobota et al., 2007) or the number of presynaptic inputs (Hou et al., 2011), and that MAG photoswitches likely do not activate receptors at the labeling concentrations used (Volgraf et al., 2006). Consistent with these previous controls, *in vivo* firing rates were similar in cortical regions expressing LiGluR or GFP. In the future, it will be important to complete a characterization of the synaptic effects of LiGluR expression, labeling, and activation.

Here we focused on the neocortex because of its importance as an integrating circuit in the nervous system and since it is a context where glutamate transmission is prominent. LiGluR photoactivation could induce more than a 10-fold increase in firing rate with rapid onset and offset (as fast as ~30 ms; average ~100 ms). Photoswitching was highly reversible and repeatable over many trials. Importantly, mice injected with GFP viruses followed by MAG injection showed no light response. LiGluR-mediated photoswitching was seen throughout all layers of the cortex, indicating that this approach will likely work throughout all regions of the mammalian brain where optical fibers can be implanted. The success of LiGluR *in vivo* will pave the way for further application of PTL-based tools in order to probe specific proteins in circuit function and higher-level brain activity. Baseline firing rates varied greatly between neurons regardless of the experimental protocol, representative of the many cortical neuronal types. As illustrated above, LiGluR-mediated photoactivation modulated the activity of both low-firing (Figure 4) and fast-firing neurons (Figure 6). Combined with the ability to manipulate a high percentage (60%) of cells within a given cortical region, these data indicate that LiGluR-mediated photoactivation is likely robust enough for behavioral manipulations, as well as cell type-specific interrogation of neuronal circuits.

We find that both dual-color “regular” MAG photoswitches and single-color MAG_460_ photoswitches may be employed in cultured neurons and *in vivo* with comparable efficacy. These two families of photoswitches provide distinct advantages that allow for the appropriate adaptation to the given application. Regular MAGs offer bistability, which is optimal for high time resolution or long time scale experiments. Furthermore, owing to their stable photostationary states, regular MAGs are highly light sensitive and largely intensity-independent. The high sensitivity of regular MAGs also compensates any trade-offs due to the poor tissue penetration of 380 nm light. On the other hand, L-MAG0_460_ photoswitches offer the simplicity of one color photocontrol, which is more easily compatible with many illumination systems and may allow for easier complexing with other optical tools. The intensity-dependence of L-MAG0_460_ allows for photoswitching effects to be titrated by adjusting the laser intensity. In addition, MAG_460_ photoswitches, unlike regular MAGs, may also be activated via two-photon infrared-light illumination, which provides high spatial precision and enhanced tissue penetration (Izquierdo-Serra et al., 2014; Carroll et al., 2015). Recent experiments have also established activation of LiGluR with red-light using tetra-*ortho*-chloro-substituted MAGs (Rullo et al., 2014). Ultimately, the ability to manipulate LiGluR photoactivation with either regular L-MAGs or L-MAG0_460_ while modulating a variety of parameters (wavelength, light intensity, etc.) allows one to mimic the properties of pharmacological compounds with enhanced subtype specificity while taking advantage of the enhanced spatial, temporal, and genetic control of optogenetics.

A key feature of the PTL-based approach is that genetic changes may be made to the protein of interest while maintaining the ability to specifically manipulate it through photoswitch conjugation. For example, one may make mutations to various functional or regulatory sites within LiGluR without disrupting its ability to become photo-activated by MAG, thus enabling direct testing of the role of the mutated residues in receptor function. This represents a major improvement in terms of temporal and spatial control over classical knock-in approaches. In this study we engineered a new LiGluR variant with decreased Ca^2+^ permeability [“LiGluR(R)”] by mutating a single key pore residue. Photoswitching of LiGluR(R) in neurons will allow one to test the important of Ca^2+^ influx, as opposed to membrane depolarization alone, in mediating the downstream, physiological effects of GluK2. Similar mutational approaches may be applied to make LiGluR variants with altered activation and desensitization kinetics, phosphorylation sites, or other properties based on the vast literature on kainate receptors.

In a similar vein we also used point mutations to engineer LiGluR variants to respond only to light without the potentially confounding effects of glutamate. LA-LiGluR and ULA-LiGluR show photoswitching comparable to LiGluR despite a decrease in apparent glutamate affinity of ~10- or 200-fold, respectively. Despite its lower glutamate affinity, GluK2 (K478A) was shown to desensitize as completely as wild-type and to have only slightly faster kinetics of deactivation, desensitization, and recovery (Weston et al., 2006). This result indicates that the glutamate moiety of MAG is in a high local concentration, as previously proposed, allowing it to maintain efficacy in the mutated variants. These low affinity LiGluR variants are an important addition to the toolset for use in physiological systems in which overexpression of glutamate receptors and endogenous glutamate release may affect synaptic strength, since they allow LiGluR photoactivation to remain orthogonal to native signaling. These newly-developed LiGluR variants complement other engineered LiGluR variants, such as GluK2 (G486C), which is activated by the *trans*-isomer of MAG rather than the *cis* isomer (Numano et al., 2009) and to the potassium selective LiGluR “HyLighter,” which can be used to hyperpolarize neurons (Janovjak et al., 2010). Together, these variants constitute a LiGluR toolset that may be further enhanced by combination of GluK2 mutants with different versions of L-MAG (Table 1).

**Table 1:** **Summary of LiGluR variants**.

**A. MAG Photoswitch**			
**Name**	**Photoswitching**	**Key features**	**References**
L-MAG0 L-MAG1 L-MAG2	*cis*: 370–395 nm *trans:* 460–540 nm	Series of bistable photoswitches with different linker lengths that turn LiGluRs *on* and *off* with light of different wavelengths; thermal relaxation occurs on slow timescales (τ = 26 min) rendering switching bistable	Numano et al., 2009
L-MAG0_460_	*cis*: 440–480 nm *trans:* darkness	Single-wavelength photoswitch (blue-light activated) with fast thermal relaxation (τ_mean_ = 0.71 s); also activated by white light and 2-photons (850 nm)	Kienzler et al., 2013; Izquierdo-Serra et al., 2014; Carroll et al., 2015
t*o*Cl-MAG1	*cis*: ~ 380 nm, >540 nm *trans:* ~ 440 nm	Activated with UV, yellow and red light (but slower kinetics at comparable light intensities); bistability through slow thermal relaxation (τ = 5 h)	Rullo et al., 2014
**B. Engineered Receptor**			
**Name**	**Mutations**	**Key features**	**References**
LiGluR	L439C	General light activated glutamate receptor based on GluK2(Q)^*^; *cis*-MAGs cause receptor activation, which results in depolarization	Volgraf et al., 2006; Gorostiza et al., 2007
LA-LiGluR	L439C K487A	Reduced sensitivity to glutamate (EC_50_~ 0.70 mM) while MAG photoswitching is retained	This study
ULA-LiGluR	L439C E738D	Strongly reduced sensitivity to glutamate (EC_50_~ 10 mM) while L-MAG1 photoswitching is retained	This study
LiGluR(R)	L439C Q621R	Low Ca^2+^ permeability (Q/R editing site in GluK2)	This study
*trans*-LiGluR	G486C	Reversed mode of action (*trans*-activated with L-MAG0)	Numano et al., 2009
Hylighter	P0-C chimera L439C	K^+^-selective LiGluR for hyperpolarization/silencing	Janovjak et al., 2010

*Versatile LiGluR toolset provided by combining (A) different MAG photoswitches with (B) engineered receptors variants*.

* *standard LiGluR is based on the GluK2a isoform with Q621 (unedited)*.

Opsin-based optogenetic tools, like channelrhodopsin and light-driven ion pumps, are widely used to control neuronal function. In these cases, the expression of only a single, relatively small construct allows for basic, yet robust control of neuronal excitability by de- or hyper-polarization of selected cells, since retinal is readily available in most model organisms. PTL-based approaches, in contrast, are based on the application of synthetic photoswitches to target genetically engineered receptor constructs. While the labeling of receptors with a PTL has to be optimized in each preparation and controls on specificity have to be included, this approach offers a number of distinct advantages. Importantly, the PTL-based approach allows for the control of signaling proteins native to the synapse. This allows one to manipulate more specific functions compared to simply altering membrane potential. For example, recent studies have used a photoswitchable mGluR2 to control presynaptic inhibition in hippocampal neurons (Levitz et al., 2013) and used a photoswitchable mGluR3 to manipulate glutamate transport in astrocytes (Li et al., 2015). Thus, in comparison to classical optogenetic approaches this PTL-based strategy does not only allow one to control cellular excitability, but to probe the function of specific receptor subtypes and their contribution to behavior with unprecedented resolution. This should help in overcoming limitations of transgenic and pharmacological approaches currently used to study the function of different glutamate receptor subtypes in different circuits. The principles of this study will be widely applicable to other PTL-based photoswitchable proteins such as metabotropic glutamate receptors (Levitz et al., 2013), potassium channels (Banghart et al., 2004, 2006; Fortin et al., 2011; Sandoz et al., 2012; Sandoz and Levitz, 2013), P2X receptors (Lemoine et al., 2013; Browne et al., 2014), GABA_A_ receptors (Lin et al., 2014, 2015), and nACh receptors (Tochitsky et al., 2012). In addition, the recent development of a SNAP-based photoswitch labeling scheme that is orthogonal to maleimide and can be used to control mGluRs (Broichhagen et al., 2015), will further expand the ability to apply these tools in experiments of increasing sophistication, including those where multiple receptors are controlled within the same preparation. Combination of these molecular approaches to optical control with knock-in or CRISPR-mediated genetic manipulation (Incontro et al., 2014; Platt et al., 2014), holds great promise for a developing a powerful new way to study the underlying molecular events that mediate brain function.

## Author contributions

JL, AP, AR, and EI designed the research and wrote the paper. JL, AP, and AR performed experiments and analyzed data.

### Conflict of interest statement

The authors declare that the research was conducted in the absence of any commercial or financial relationships that could be construed as a potential conflict of interest.
