# Supplementary material for: A Toolkit for Orthogonal and in vivo Optical Manipulation of Ionotropic Glutamate Receptors
Source: Front Mol Neurosci. 2016 Feb 2;9:2. doi: 10.3389/fnmol.2016.00002 (PMC4735401; doi:10.3389/fnmol.2016.00002)
Supplement: Supplementary file 1 [file SupplementaryMaterial.pdf]

Supplementary Material for:

A Toolkit for Orthogonal and *in vivo* Optical Manipulation of Ionotropic Glutamate Receptors

Joshua Levitz[1,#], Andrei T. Popescu[1,#], Andreas Reiner[1,2,#], Ehud Y. Isacoff[1,3,4,*]

[1] Department of Molecular and Cell Biology, University of California, Berkeley, California, 94720

[2] Department of Biology and Biotechnology, Ruhr-University Bochum, Bochum, Germany, 44801

[3] Helen Wills Neuroscience Institute, University of California, Berkeley, California, 94720

[4] Physical Bioscience Division, Lawrence Berkeley National Laboratory, Berkeley, California, 94720

*To whom correspondence should be addressed: ehud@berkeley.edu

# Equal contribution

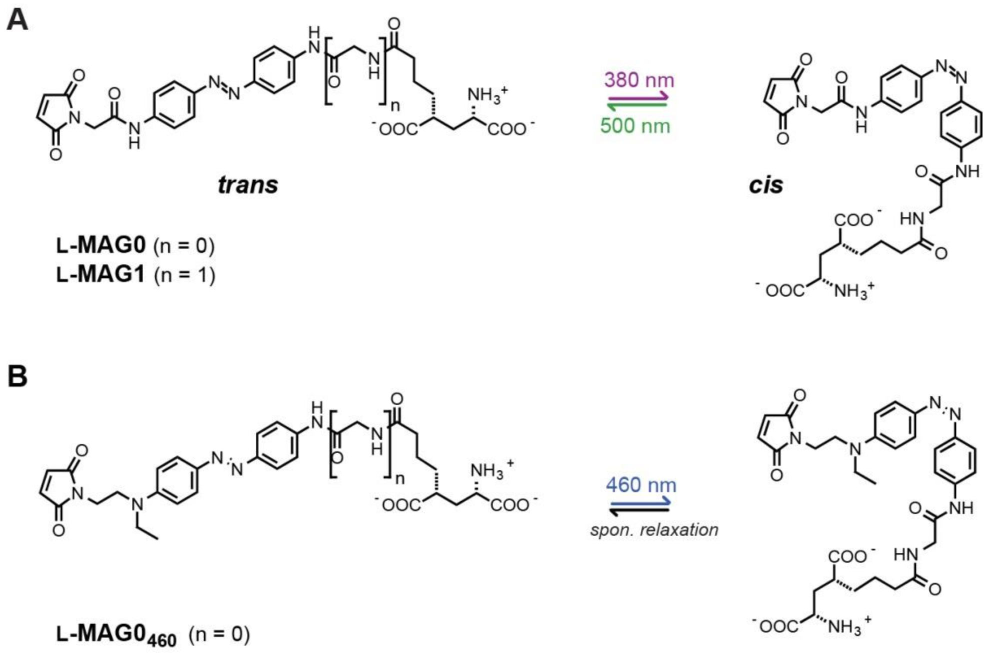

Fig. S1. Chemical structure of MAG photoswitches used in this study.
(A) Regular L-MAG0 and L-MAG1 differ in the presence of a glycine spacer between the central azobenzene moiety and the 4-alkylglutamate headgroup. **(B)** L-MAG0$_{460}$ encompasses a “push-pull” azobenzene core, which can be operated as a single-wavelength blue light photoswitch.

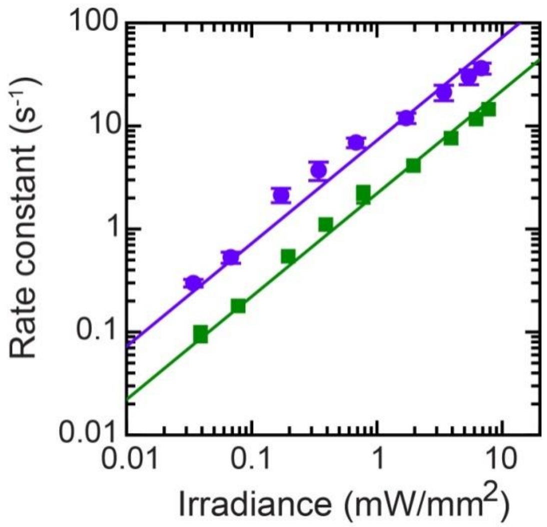

Fig. S2. Activation and deactivation kinetics of LiGluR conjugated with L-MAG0. At light intensities typical for optogenetic experimentation the apparent activation and deactivation kinetics of LiGluR are determined by the light intensity (HEK cell recording, see Fig. 1). The kinetics are well described by single exponential functions yielding rate constants of photo-activation (violet, 380 nm) and photo-deactivation (green, 500 nm) as a function of the irradiance (light intensity/area; mean ± s.d., $n = 5$ cells). The relationships are linear with sensitivities (linear slopes) of 7.3 s^{-1} $(mW/mm^2)^{-1}$ for 380 nm and 2.2 s^{-1} $(mW/mm^2)^{-1}$ for 500 nm, respectively. Very high light intensities can be used to drive LiGluR on the submillisecond timescale (Reiner et al., 2014b), which is not shown here. The behavior of LiGluR conjugated with L-MAG0$_{460}$ was reported in (Gaub et al., 2014).

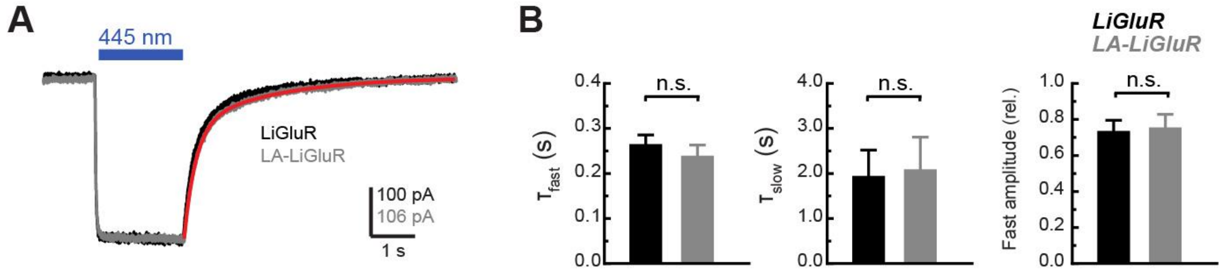

Fig. S3. Spontaneous relaxation of LiGluR and LA-LiGluR labeled with L-MAG0$_{460}$.
(A) Overlay of the spontaneous relaxation of LiGluR (black) and LA-LiGluR (grey) after the 445 nm light (blue bar) is turned off. The red curves represents a double exponential function used for fitting. **(B)** Relaxation parameters, namely fast time constant τ_{fast}, slow time constant τ_{slow}, and the rel. amplitude distribution between τ_{fast} and τ_{slowt} obtained with double exponential fits (means ± s.d.; LiGluR *n* = 3 cells, LA-LiGluR *n* = 6 cells). T-tests did not detect any statistically significant differences in the relaxation behavior.

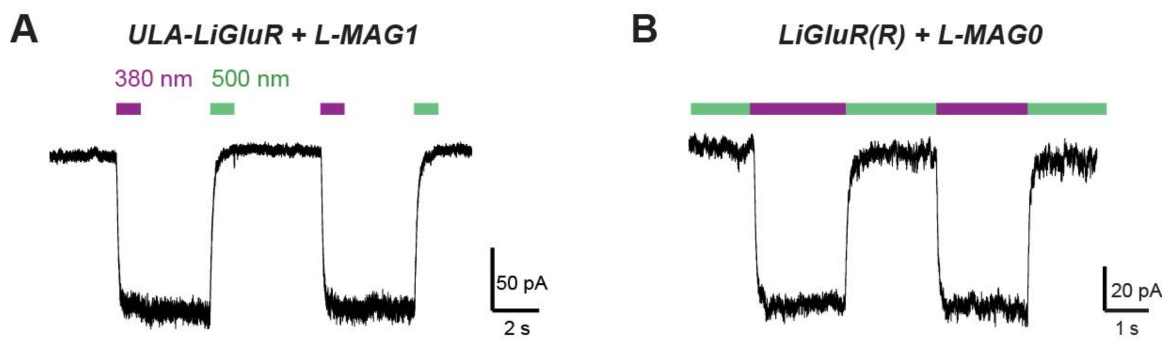

Fig. S4. Photoswitching of ULA-LiGluR and LiGluR(R).

Voltage-clamp recordings from HEK293 cells expressing **(A)** ULA-LiGluR labeled with L-MAG1 and **(B)** LiGluR(R) labeled with L-MAG0. Illumination was performed with a DG4 light source with an irradiance of 1- 2 mW/mm^2. The violet and green bars indicate 380 nm and 500 nm illumination periods, respectively. For details see Material and Methods.

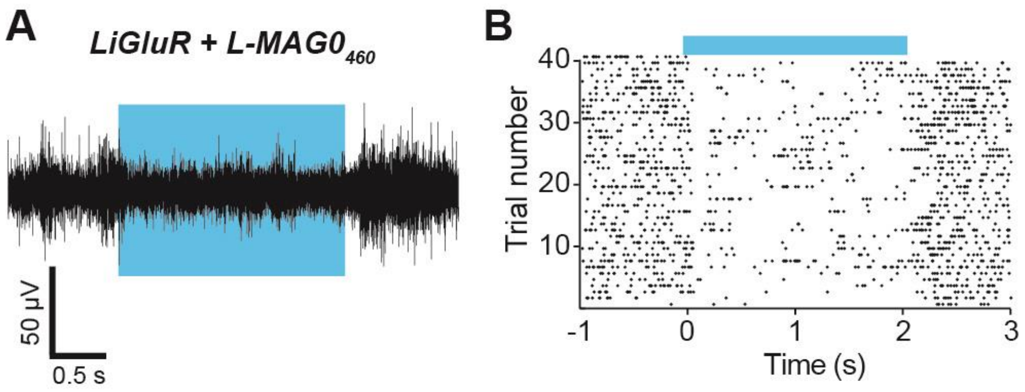

Fig. S5. *in vivo* inhibition in response to LiGluR photoactivation.
Example of a multi-synaptic effect of LiGluR activation through feed-forward inhibition in the local cortical circuit. **(A)** A high-pass recording showing inhibition of neuronal firing in response to laser stimulation. **(B)** Raster plot of the same neuron, illustrating each action potential (individual dots) as a function of time (x-axis) in response to individual laser pulses (y-axis).
